# Shifting Paradigms for Suppressing Fibrosis in Kidney Transplants: Supplementing Perfusion Solutions With Anti-fibrotic Drugs

**DOI:** 10.3389/fmed.2021.806774

**Published:** 2022-01-10

**Authors:** L. Leonie van Leeuwen, Henri G. D. Leuvenink, Peter Olinga, Mitchel J. R. Ruigrok

**Affiliations:** ^1^Department of Surgery, University Medical Center Groningen, University of Groningen, Groningen, Netherlands; ^2^Department of Pharmaceutical Technology and Biopharmacy, Groningen Research Institute of Pharmacy, University of Groningen, Groningen, Netherlands

**Keywords:** renal transplantation, machine perfusion, IRI, donation after circulatory death (DCD), personalized medicine, IF/TA, precision cut tissue slices

## Abstract

Great efforts have been made toward addressing the demand for donor kidneys. One of the most promising approaches is to use kidneys from donation after circulatory death donors. These kidneys, however, suffer from more severe ischemia and reperfusion injury than those obtained *via* donation after brain death and are thus more prone to develop interstitial fibrosis and tubular atrophy. Even though machine perfusion is increasingly used to reduce ischemia and reperfusion injury, there are no effective treatments available to ameliorate interstitial fibrosis and tubular atrophy, forcing patients to resume dialysis, undergo re-transplantation, or suffer from premature death. Safe and effective anti-fibrotic therapies are therefore greatly desired. We propose a new therapeutic approach in which machine perfusion solutions are supplemented with anti-fibrotic compounds. This allows the use of higher concentrations than those used in humans whilst eliminating side effects in other organs. To the authors' knowledge, no one has reviewed whether such an approach could reduce interstitial fibrosis and tubular atrophy; we therefore set out to explore its merit. In this review, we first provide background information on ischemia and reperfusion injury as well as interstitial fibrosis and tubular atrophy, after which we describe currently available approaches for preserving donor kidneys. We then present an evaluation of selected compounds. To identify promising compounds, we analyzed publications describing the effects of anti-fibrotic molecules in precision-cut kidneys slices, which are viable explants that can be cultured *ex vivo* for up to a few days whilst retaining functional and structural features. LY2109761, galunisertib, imatinib, nintedanib, and butaprost were shown to exert anti-fibrotic effects in slices within a relatively short timeframe (<48 h) and are therefore considered to be excellent candidates for follow-up *ex vivo* machine perfusion studies.

## Introduction

**Figure 1:**
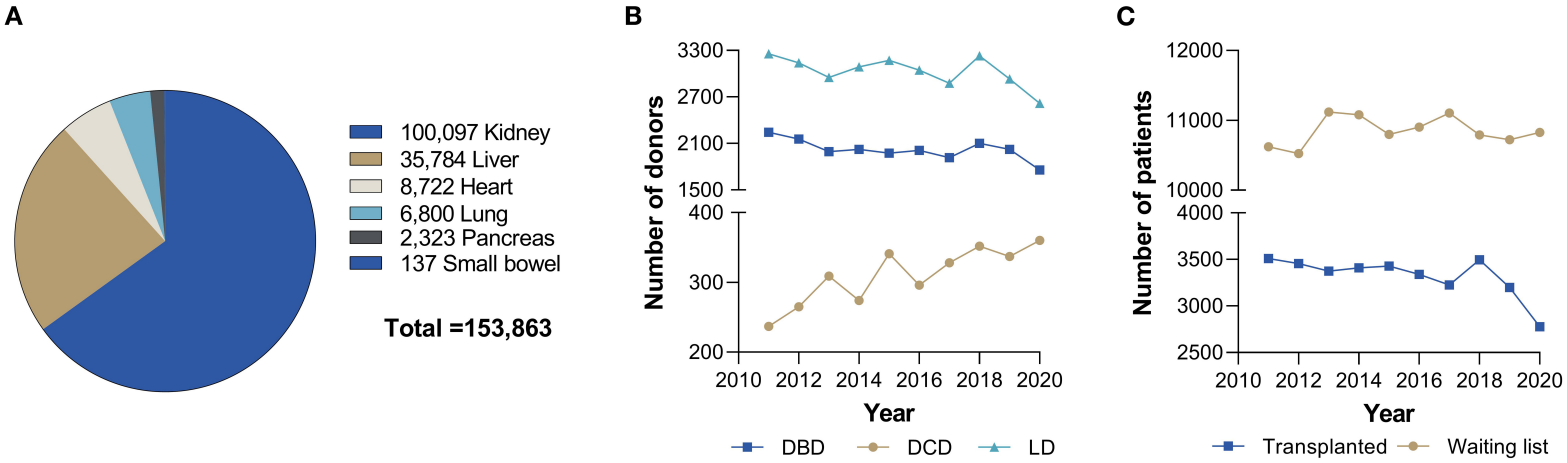
**(A)** Total number of organs transplanted worldwide. **(B)** Number of donors per donor type within Eurotransplant over the past 10 years. **(C)** Number of kidney transplants and active waiting list for a kidney transplant within Eurotransplant over the past 10 years. Data obtained from Global observatory on donation and transplantation in the year 2019 (6) and Eurotransplant (7). DBD, donation after brain death; DCD, donation after circulatory death; LD, living donor.

Kidney transplantation is a life-saving procedure that significantly improves the lives of patients who suffer from end-stage renal disease, which is characterized by glomerulosclerosis and tubulointerstitial fibrosis and represents the last stage of chronic kidney disease (1–3). The increase in life span conferred by kidney transplantation has been estimated to be ~10 years, and ranges from 3 to 17 years depending on patient group (4). Kidneys are the most frequently transplanted organ in the world, with over 100,000 transplantations in 2019 (Figure 1A) (5). Organs for kidney transplantation are predominantly obtained through donation after brain death (DBD), although other sources exist as well, including the use of kidneys from living donors (Figure 1B) (6). Still, there remains a tremendous demand for donor kidneys, far exceeding their supply (Figure 1C). Patients therefore have to wait several years before receiving a donor kidney (1, 4).

In recent years, immense efforts have been focused toward addressing the demand for donor kidneys. One of the most promising approaches is to use kidneys from donation after circulatory death (DCD) donors (Figure 1B) (6, 8–10). DCD kidneys are obtained from donors that do not meet the standard criteria for brain death and whose cardiac function ceased either spontaneously (e.g., due to cardiac arrest in the emergency room) or deliberately (e.g., upon withdrawing life support systems) (11). In comparison to DBD kidneys, DCD kidneys are more likely to develop ischemia and reperfusion injury (IRI), possibly resulting in less favorable clinical outcomes. As a result, short-term complications, such as delayed graft function (DGF) or early graft failure, are observed more frequently for these kidneys (12, 13). Some clinicians are thus reluctant to accept DCD kidneys. Emerging evidence, however, indicates long-term clinical outcomes of DCD kidneys are comparable to those for DBD kidneys (6).

The most common cause for kidney allograft failure is chronic allograft nephropathy, which is characterized by interstitial fibrosis (IF) and tubular atrophy (TA) (14). This disorder leads to a gradual decline in kidney function as well as hypertension and proteinuria. At the core of IF lies dysregulated wound-healing, driven by chronic injury and inflammation, favoring the deposition of extracellular matrix (ECM) proteins, including collagens, fibronectins, and many more (15, 16). Chronic injury also promotes the development of TA—a condition marked by flattening of tubular epithelial cells, contraction of the tubular lumens, and thickening and wrinkling of tubular basement membranes (15). The drivers of IF/TA are not always clear, but it is known to occur already during the first few years after transplantation. Unfortunately, recipients of kidney transplants who develop IF/TA are forced to eventually resume dialysis, undergo re-transplantation, or suffer from premature death (12, 17). As there are currently no effective treatments available to suppress or prevent IF/TA, there is a great medical need for more effective and safer therapies.

Despite the lack of anti-fibrotic therapies, there are approaches available to reduce damage in donor kidneys, the most effective one being the use of machine perfusion—an increasingly popular method to preserve grafts, especially those obtained from suboptimal donors such as DCD donors (18). This technique could be further improved by adding anti-fibrotic drugs to perfusion solutions, reducing the risk of systemic side effects. To the authors' knowledge, no one has reviewed whether such an approach could reduce IF/TA in kidney transplants; we therefore set out to explore its merit. In this review, we first provide background information on the sources of damage in kidney transplants and the development of IF/TA, after which we describe currently available approaches for preserving donor kidneys. We then present an evaluation of anti-fibrotic compounds that appear promising for use during machine perfusion, followed by an outline of recommendations for future research to accelerate developments. We have to be swift to realize this paradigm shift.

## Ischemia and Reperfusion Injury

**Figure 2:**
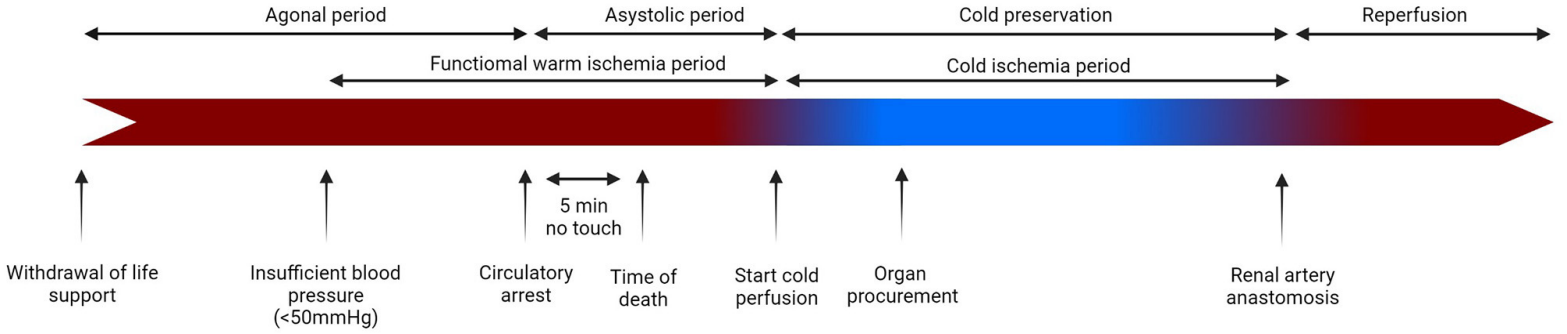
The process of donation after circulatory death, organ procurement, and transplantation. Created with BioRender.com.

To understand the complex pathophysiological processes underlying chronic allograft nephropathy, it is essential to recognize the main source of tissue damage: IRI. During DCD donation, IRI starts immediately after withdrawing life support (Figure 2). This causes the donor to enter a withdrawal (agonal) period of hemodynamic instability until cardiac function ceases. At some point during this process, the blood pressure becomes insufficient to support organ function, starting the so-called “functional” warm ischemia time. Circulatory arrest follows, marking the start of the “standard” warm ischemia time, which extends until the onset of cold *in situ* perfusion of abdominal organs (19, 20). After surgical removal, donor kidneys are stored and transported in ice-cold preservation solution. Cold preservation reduces, but does not eliminate, cellular injury (21). In fact, prolonged warm and cold ischemia is associated with DGF, graft failure, and mortality after kidney transplantation (22–25). Upon transplantation, the graft is anastomosed to the arteries of the recipient, establishing reperfusion. Reperfusion, however, does not directly restore physiological conditions. As a matter of fact, it initially exacerbates damage caused by ischemia.

### Ischemia

**Figure 3:**
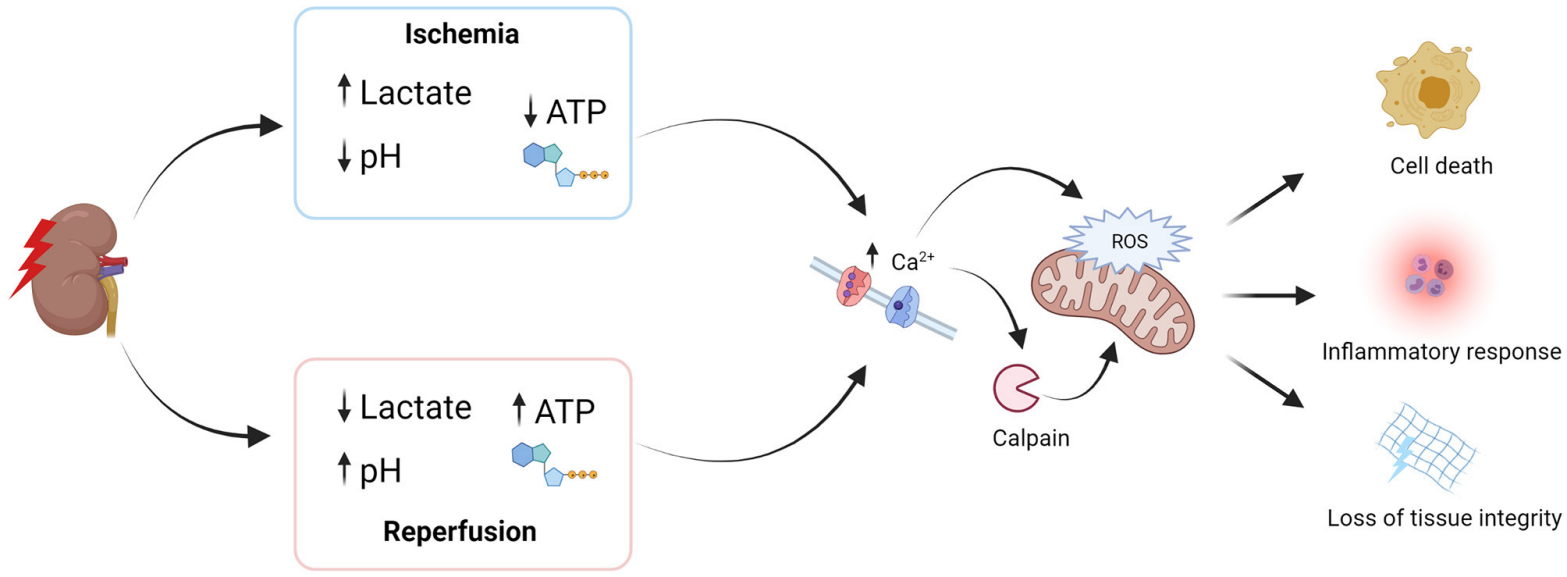
Ischemia and reperfusion injury as a result of donation after circulatory death. ATP, adenosine triphosphate; ROS, reactive oxygen species. Created with BioRender.com.

Ischemia occurs when the blood supply to tissue becomes restricted, causing a shortage of oxygen that is needed for the production of adenosine triphosphate (ATP). As a result of ischemia, cells switch from highly efficient aerobic respiration (i.e., 29–30 ATP molecules per glucose molecule) to far less efficient anaerobic respiration (i.e., 2 ATP molecules per glucose molecule) (26, 27). This causes a decline in intracellular ATP levels and increased levels of lactate, which contribute to intracellular acidosis and cell membrane injury (Figure 3) (28). ATP depletion also affects intracellular calcium (Ca^2+^) levels because Ca^2+^ pumps, which are ATP-dependent, cannot pump Ca^2+^ out of cells (29). Increased levels of intracellular Ca^2+^ subsequently cause activation of various calcium-dependent enzymes, such as calpains, endonucleases, and phospholipases. In turn, these enzymes contribute to the generation of reactive oxygen species (ROS) and the breakdown of the cell membrane, among other processes (30). It is therefore unsurprising that epithelial cells, in particular proximal tubular cells, are highly susceptible to ischemia, given the fact that these cells require a lot of ATP for mediating ion transport (31).

### Reperfusion

Upon reperfusion, the blood supply is restored, and the ischemic environment induces an inflammatory response. The re-introduction of oxygen, rewarming of the kidney graft, and pH normalization is detrimental to the previously ischemic cells. Sudden increases in oxygen levels lead to extensive ROS production. The production of ROS is normally counteracted by antioxidants, but the high levels after reperfusion overwhelm this protection mechanism (32). pH normalization leads to a further increase in intracellular Ca^2+^ levels that, together with the generated ROS, further activate calcium-dependent enzymes (e.g., calpains, etc.). Activated calpains promote inflammation and damage cells by selectively degrading a large number of intracellular proteins, including signaling proteins, cytoskeletal proteins, and transcription factors (33, 34). The electron transport chain may also become unstable. This causes the formation of mitochondrial permeability transition pores which, in turn, promote the release of cytochrome C into the cytosol to activate caspases—the key effector proteins in apoptosis (30).

## Interstitial Fibrosis and Tubular Atrophy

It is clear that IRI promotes cell death by apoptosis and/or necrosis, especially in tubular and glomerular cells (35–38). Apart from that, IRI triggers an immune response, activating both the innate and adaptive immune system. IRI also affects the structural integrity of kidney tissue, as marked by swelling of endothelial cells, loss of their glycocalyx, and degradation of the cytoskeleton (39). In response to these forms of damage, molecular processes supporting wound-healing, also known as “fibrogenesis,” are activated in an attempt to restore tissue integrity and function.

### Normal Wound-Healing: Fibrogenesis and Epithelial Dedifferentiation

**Figure 4:**
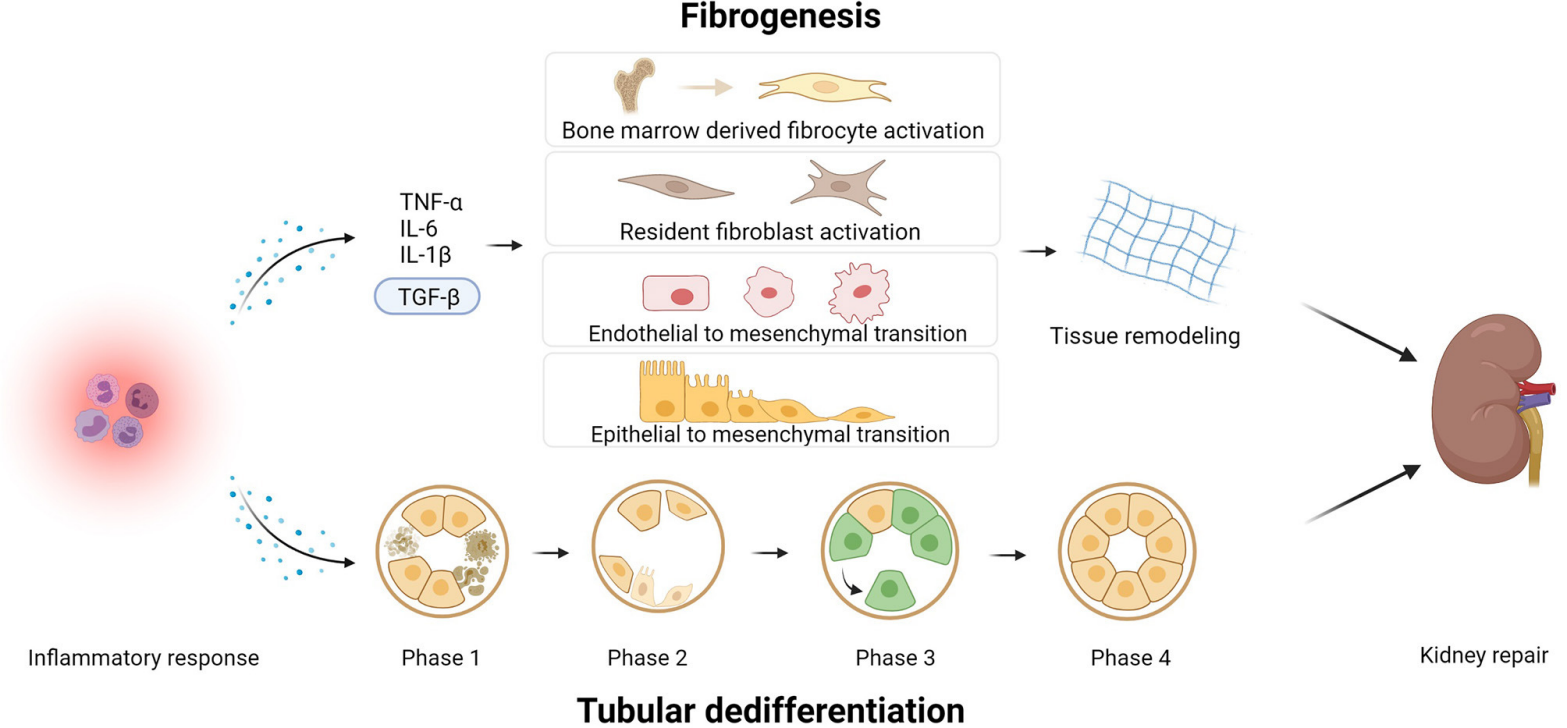
Kidney repair after ischemia and reperfusion injury by means of fibrogenesis and tubular dedifferentiation. TNF-α, tumor necrosis factor; IL-6, Interleukin; IL-β1, interleukin 1β; TGF-ß, transforming growth factor. Created with BioRender.com.

Fibrogenesis represents a well-coordinated, multicellular regenerative response that is triggered upon injury (Figure 4). When renal tissue gets injured, an inflammatory response is elicited, involving various immune cells, such as T-cells, dendritic cells, macrophages, and neutrophils (40). Triggered by inflammatory cytokines, including but not limited to tumor necrosis factor alpha (TNF-α), interleukin 1 beta (IL-1β), interleukin 6 (IL-6), and transforming growth factor beta 1 (TGF-β1), these immune cells are recruited to affected sites and become activated to remove tissue debris as well as dead and damaged cells (41). TGF-β1 in particular also promotes the accumulation of myofibroblasts, which are key effector cells in wound-healing. Myofibroblasts have been shown to arise from various sources, namely: proliferation and differentiation of resident interstitial fibroblasts (50%), infiltration and differentiation of fibrocytes (35%), endothelial-to-mesenchymal transition (EndoMT) (20%), and epithelial-to-mesenchymal transition (EMT) (5%) (42–45). The extent of EndoMT and EMT, however, remains controversial and it is currently unclear whether these processes plays a substantial role in humans. Once myofibroblasts become activated, characterized by *de novo* expression of alpha smooth muscle actin (α-SMA), these cells aim to restore tissue integrity by producing and secreting ECM proteins, especially collagens and fibronectins (43).

The provisional, collagen-rich matrix provides a scaffold for cell growth (e.g., to facilitate tubular regeneration). In normal conditions, the renewal rate of tubular epithelial cells is low. This rate, however, drastically increases after (ischemic) injury, with the aim of restoring the tubular epithelium via dedifferentiation (46). Indeed, fully differentiated tubular epithelial cells have been shown to repair proximal tubules without the contribution of intralobular stem cells. This process comprises four stages. Stage one is characterized by apoptosis or necrosis of damaged tubular epithelial cells as well as inflammation. In the second stage, tubular cells flatten and lose their brush border as well as polarity. EMT has also been shown to occur in this stage. Stage three is marked by increased secretion of growth factors, which stimulate tubular epithelial cells to enter the cell-division cycle. In the last stage, the tubules become repopulated with daughter-cells, and morphological features of tubular structures recover (46). After these processes, kidney function is restored.

### Wound-Healing Gone Rogue: Interstitial Fibrosis and Tubular Atrophy

**Figure 5:**
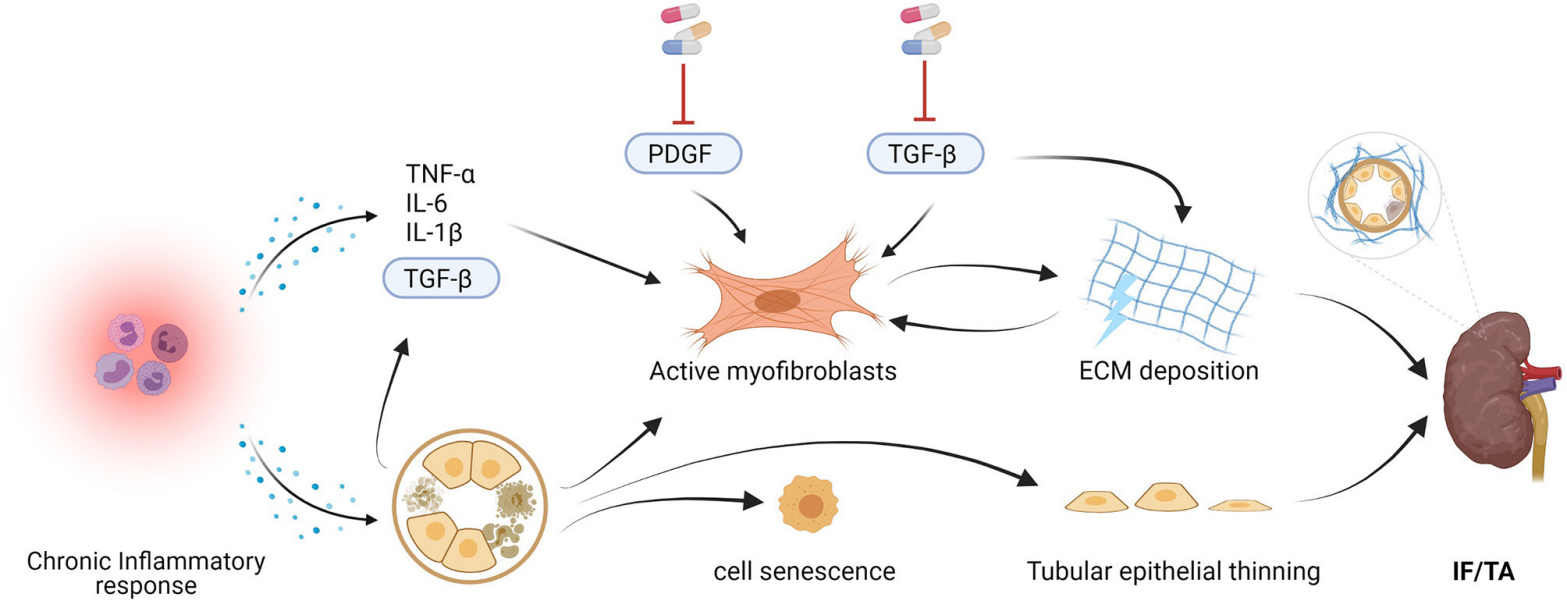
Chronic injury and development of interstitial fibrosis and tubular atrophy (IF/TA). Continuous activation of myofibroblasts cause excessive deposition of extracellular matrix (ECM). Given enough time, the dense, fibrotic ECM perpetuates myofibroblast activation. Tubular dedifferentiation is impeded by cell senescence and apoptosis. Pathways such as the platelet-derived growth factor (PDGF) and the transforming growth factor (TGF-ß) SMAD2/3 pathway play a key role in myofibroblast activation and therefore represent promising drug targets. TNF-α, tumor necrosis factor; IL-6, Interleukin 6; IL-1β, interleukin 1β. Created with BioRender.com.

In some cases, the wound-healing process becomes severely dysregulated, resulting in the uncontrolled deposition of ECM proteins into the intertubular, extraglomerular, extravascular space of the kidney (Figure 5) (47, 48). This phenomenon is called “interstitial fibrosis.” Fibrosis is a disorder in which the excessive deposition of ECM proteins afflict the kidney architecture and function. The root-cause of the shift from fibrogenesis to fibrosis remains unclear. Some aspects, however, have been shown to play a role, such as the persistence and duration of injury. Chronic injury, for example, contributes to the unbridled recruitment of immune cells as well as activation of myofibroblasts. Given enough time, the ECM changes with respect to its composition (i.e., collagen and fibronectin-rich), organization (i.e., densely crosslinked fibers), and stiffness. In fibrosis, these changes to the ECM are thought to have passed a point-of-no-return, wherein the ECM itself starts to perpetuate myofibroblast activation. One of the key mediators during this pathophysiological process is TGF-β, which binds to its cell membrane type I and II receptor, causing receptor phosphorylation and so activation of its downstream signaling mediators SMAD2 and SMAD3 (Figure 5) (49).

Interstitial fibrosis is often observed together with tubular atrophy, as both pathologies can be the result of IRI (50). TA is characterized by the disappearance and/or flattening of tubular epithelial cells, contraction of tubular lumens, and thickening as well as wrinkling of tubular basement membranes (Figure 5) (15). Unsurprisingly, these processes reduce the tubular reabsorption of water and small molecules. Like fibrosis, the pathogenesis of TA involves various mechanisms, of which the extent of involvement has not been fully elucidated. Apoptosis, for example, could become persistent, effectively reducing the number of cells as a result of (chronic) injury (50). Schelling have also observed increased levels of senescence in remaining epithelial cells (50). Senescent cells have lost the ability to divide and therefore further impede tubular dedifferentiation. Sometimes, chronic histological damage is already visible as early as 3 months post-transplantation (51).

## Current Strategies for Preserving Donor Kidneys

**Figure 6:**
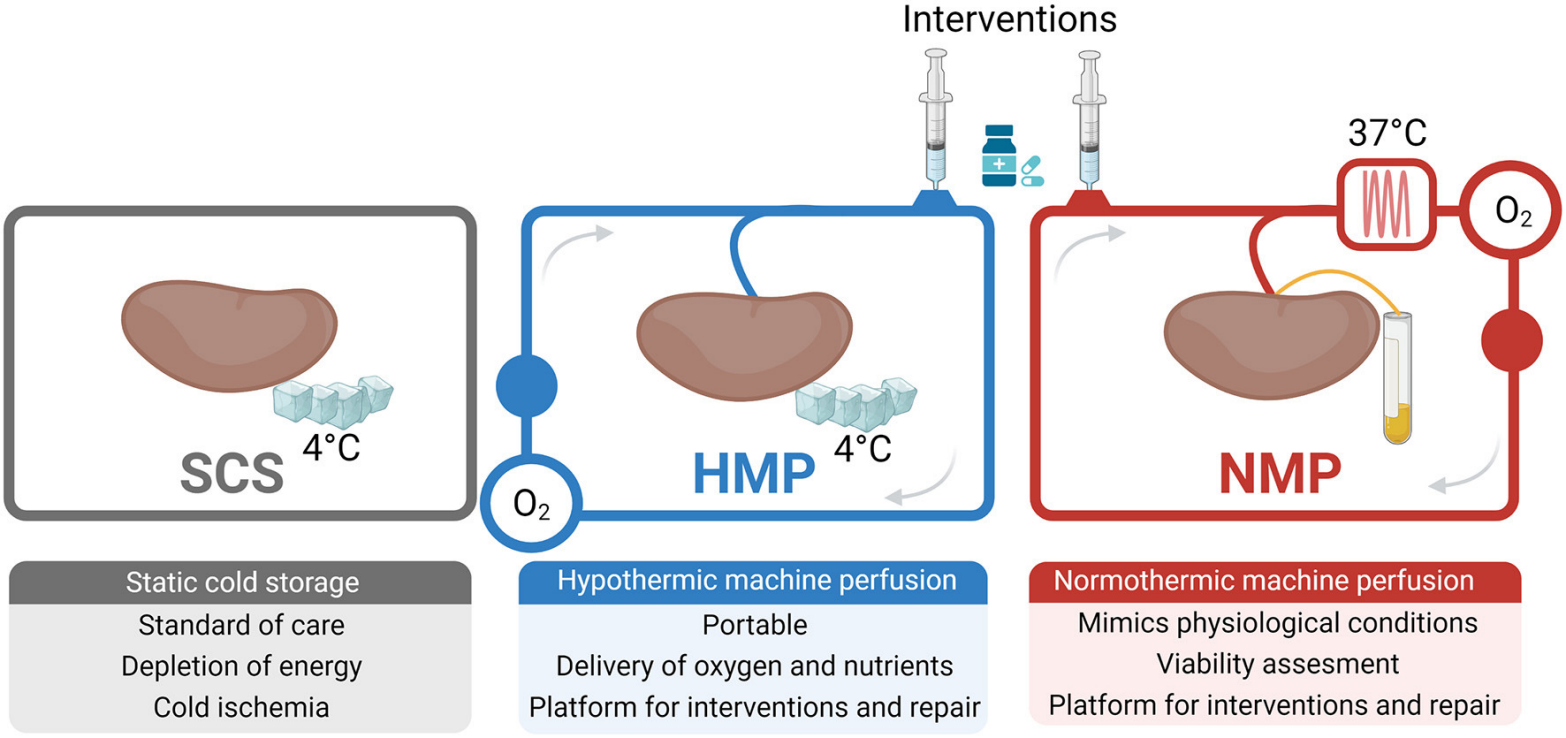
Current strategies for preserving donor kidneys. Created with BioRender.com.

Throughout the years, various preservation techniques have been developed to facilitate the storage and transport of donor organs (Figure 6). Static cold storage (SCS) is the most commonly applied preservation technique and has been the accepted clinical standard for organ preservation in most countries since the 1960s (52). The past decade, however, has revealed the benefits of using machine perfusion. In fact, the use of machine perfusion for preserving deceased donor kidneys is the current standard of clinical care in The Netherlands, as it shows better outcomes compared to SCS (53).

### Static Cold Storage

SCS is the cheapest and simplest technique for preserving donor kidneys. When using this approach, kidney grafts are first flushed with ice-cold preservation solution and then stored on ice until further use (52, 54). So far, great efforts have been put into the development of preservation solutions, each with the purpose of maintaining the viability of grafts. The benefits and drawbacks of various solutions are discussed in an extensive review written by Hosgood et al. (55). Nonetheless, SCS suffers from several limitations, such as the fact that hypothermic and ischemic conditions as well as nutritional deficiencies do not fully stop cellular respiration or prevent the formation of metabolic waste products (e.g., lactate). As a result, cells switch to anaerobic respiration, depleting energy reserves and leading to acidification of the cellular environment (52, 54, 56). The cold ischemia time (CIT) is therefore directly associated with DGF (21, 23). Each additional hour of CIT, for example, significantly increases the risk of graft failure and mortality, especially for grafts from extended criteria and DCD donors (24, 57).

### Hypothermic Machine Perfusion

Hypothermic machine perfusion (HMP) has been introduced as an alternative to SCS because it offers superior organ preservation, especially for grafts of suboptimal quality. HMP refers to the controlled flow of perfusion solution through grafts at a temperature of 4°C (58). University of Wisconsin machine perfusion (UW-MP) solution is used most often. UW-MP contains nutrients and metabolites to support the reduced cellular metabolism during HMP (59). It has been shown that HMP protects endothelial cells and reduces pro-inflammatory cytokine expression (e.g., TNF-α and IL-1β) (60–62). In a seminal study, Moers et al. (63) described that, in comparison to SCS, HMP led to reduced serum creatinine levels post-transplantation and a reduced risk of graft failure. On top of that, the use of HMP significantly increased the 1-year graft survival rate compared to SCS (94 vs. 90%, *p* = 0.04).

Nowadays, HMP is frequently performed using oxygenated perfusion solution, as it has been shown to promote ATP synthesis, preserve mitochondrial homeostasis and reduce interstitial fibrosis in porcine kidneys (64–66). Jochmans et al. (67) demonstrated that oxygenation (100% oxygen at 100 mL/min) lessened post-transplant complications and significantly increased the 1-year graft survival rate compared to HMP without oxygen (95 vs. 89%, *p* = 0.028). Therefore, oxygenated HMP may be a promising therapy to reduce the odds of developing IF/TA, but this will require long-term follow-up studies.

Last but not least, HMP allows for sampling of the perfusate to assess the viability, as elegantly shown by Moers et al. (68) who analyzed glutathione-S-transferase, N-acetyl-β-D-glucosaminidase, and heart-type fatty acid binding protein to predict DGF. Emerging techniques, such as proteomics, also appear promising, as reported by van Leeuwen et al. (69), who found out that the HMP perfusate composition associates with the 1-year transplant outcome.

### Normothermic Machine Perfusion

Normothermic machine perfusion (NMP) represents an advanced form of preservation. During NMP, organs are perfused with a blood-based solution at 37°C, mimicking the physiological conditions to support metabolic activity and organ function (52). The perfusate composition between NMP protocols varies greatly, as the exact metabolic needs of an isolated kidney are still not fully characterized (70, 71). Having said that, studies have revealed the importance of using an oxygen carrier (e.g., red blood cells or artificial oxygen carriers), even when supraphysiological concentrations of oxygen are used (72, 73). Short-term outcomes for porcine kidneys that were subjected to NMP have shown to be superior compared to those subjected to SCS (74–76). Studies comparing HMP and NMP have not revealed differences in transplant outcomes yet (77).

Like HMP, NMP also allows for sampling and subsequent analysis of perfusate. NMP, however, offers another benefit as it can be used to assess kidney function (i.e., urine production, creatinine clearance, and fractional sodium excretion) as metabolism is restored. Carefully monitoring graft function during NMP and measuring biomarkers in the perfusate can facilitate decision-making during transplantation (52, 78, 79). In spite of that, standardized viability criteria are lacking because no one has managed to predict post-transplant outcomes yet. Although NMP itself seems to offer superior graft preservation in comparison to SCS, supplementing perfusate with pharmacologically-active compounds may further improve the preservation of metabolically active donor kidneys.

### Suppressing the Onset of Fibrosis *ex vivo*

Current approaches for suppressing fibrosis are mostly focused on minimizing damage to kidney grafts (e.g., donor age, ischemia time, and machine perfusion). With the shortage of donor kidneys, however, these strategies are not sufficient. Supplementing NMP solutions with anti-fibrotic drugs could be a promising strategy to suppress the onset of fibrosis and, in turn, reduce graft failure. Unfortunately, research into this area is limited, as conventional models such as cell cultures and animal studies have a limited predictive value when it comes to assessing the efficacy of anti-fibrotic drugs. Cell cultures are useful for investigating cell-specific responses but do not recapitulate the intricate cell-cell and cell-matrix interactions as well as mechanical cues that are observed in fibrosis. Of course, animal models using rodents provide considerably more useful data, owing to the presence of a functional immune system and interorgan crosstalk, yet interspecies differences remain (79–81). We therefore carefully considered the models used whilst selecting relevant literature on anti-fibrotic compounds. It was also important to take into account the potency of compounds as well as the rate at which anti-fibrotic effects were produced. These factors are crucial, as the maximum NMP time is currently limited to 24 h (80).

**Figure 7:**
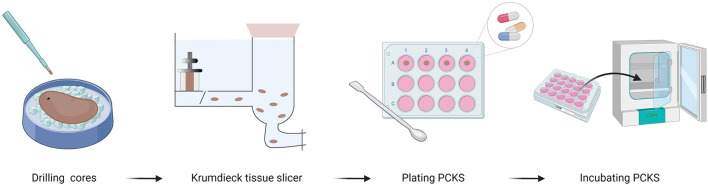
Workflow for preparing precision-cut kidney slices (PCKS). First, cores are drilled from the renal cortex and sliced using a Krumdieck tissue slicer. Next, PCKS are transferred to culture plates with fresh culture medium containing nutrients and antibiotics, as well as compounds of choice. Finally PCKS can be incubated for up to 72 h (82). Created with BioRender.com.

To identify promising anti-fibrotic compounds, we reviewed literature describing the use of precision-cut kidney slices (PCKS). PCKS are viable explants, with consistent dimensions that can be used to study fibrogenesis and fibrosis as well as the efficacy of anti-fibrotic compounds (81). The advantage of PCKS is that they contain a large number and diversity of cells, accurately reflecting the renal architecture (82). On top of that, PCKS can be prepared from (fibrotic) human tissue, creating an unprecedented opportunity to validate the molecular basis of anti-fibrotic drug candidates in humans. PCKS can also be quickly prepared in a reproducible manner by means of a specialized workflow (Figure 7). In short, cylindrical cores are prepared from kidney tissue using a biopsy puncher, after which they are placed into a Krumdieck tissue slicer and sliced under hypothermic and oxygenated conditions. PCKS are subsequently transferred to culture plates containing fresh and pre-warmed culture medium and are then incubated at 37°C, 80% O_2_, and 5% CO_2_, whilst being gently shaken (82). Taking into account the advantages and flexibility of PCKS (e.g., using different timepoints, compound concentrations etc.), it becomes clear that their use represents a valuable intermediate step before assessing anti-fibrotic compounds in an NMP setup.

### Small Molecule Drugs

**Figure 8:**
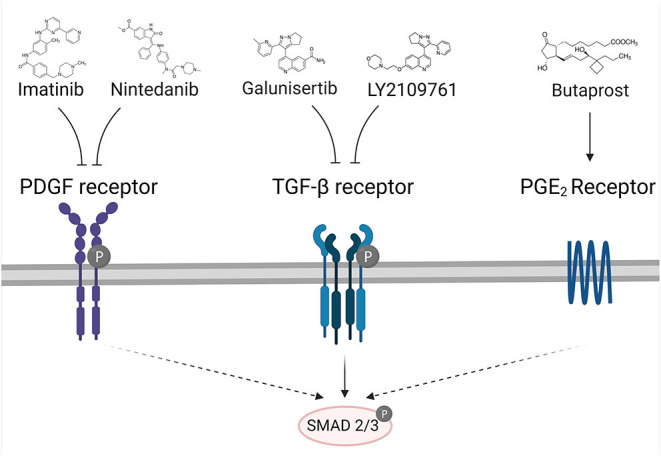
Potential compounds to limit the development of post-transplant fibrosis and their mechanism of action. PDGF: platelet-derived growth factor, TGF-β, transforming growth factor beta; PGE_2_, Prostaglandin E_2_. Created with BioRender.com.

Several drug candidates have been shown to have anti-fibrotic effects on PCKS (Table 1). Each of these small-molecule drugs produced effects already within 48 h of incubation. We hypothesize that these compounds could be promising for use during NMP, as each inhibited fibrosis via the TGF-β/SMAD signaling pathway (Figure 8). The respective studies will be discussed in this section.

**Table 1:** Potential compounds to limit the development of post-transplant fibrosis that have been tested in PCKS.

**Compound name**	**Concentrations**	**Species**	**Incubation time**	**Effect on fibrosis**	**Target**	**References**
LY2109761	5 μM	Mouse	48 h	↓	TGF-β receptor I/II	(83)
Galunisertib	10 μM	Human	48 h	↓	TGF-β receptor I/II	(84, 85)
Imatinib	10 μM	Human	48 h	↓	PDGF receptor	(85)
Nintedanib	1 μM	Human	48 h	↓	PDGF receptor	(81)
Butaprost	50 μM	Human	48 h	↓	EP_2_ receptor	(86)

#### LY2109761

LY2109761 is an oral small-molecule TGF-β receptor type I/II dual inhibitor that has been extensively studied in cancer research (87–89). Some studies, however, have shown that this molecule also reduces the extent of pulmonary and renal fibrosis (83, 90, 91). Stribos et al. (83), for example, demonstrated that LY2109761 exerted strong anti-fibrotic effects in PCKS prepared from mouse tissue, as it significantly downregulated the expression of various fibrosis-related genes (e.g., *Col1a1, Serpinh1, Fn-1, Acta-2, Serpine1*) within 48 h of incubation, without affecting the mitochondrial activity. The authors also observed a significant reduction in the expression of HSP47—a molecular chaperone that is required for collagen synthesis (92). Unfortunately, it is not known whether this compound also produced effects after 24 h of incubation because this timepoint was not included in the study. It also remains unclear whether LY2109761 has anti-fibrotic effects in PCKS prepared from human tissue. Follow-up studies should address these points as well as the lacking information with respect to side effects (e.g., the extent of cell death, effects on cell proliferation etc.).

#### Galunisertib

Galunisertib, also known as LY2157299, is an oral small-molecule inhibitor that also targets the TGF-β receptor type I/II, thereby suppressing activation of the downstream SMAD2/3 pathway (93). Galunisertib was initially developed as an antineoplastic drug (93), but has also been reported to reduce fibrosis in various mouse models and PCKS (84, 85, 94–96). For example, Bigaeva et al. (84), recently demonstrated that galunisertib downregulated mRNA expression of *Col1a1, Acta-2, Serpinh1, Fn-1, Tgfb1*, and *Tgfbr* in PCKS prepared from mouse tissue. The authors also observed a significant reduction in the expression of HSP47 and phosphorylation of SMAD2. In a follow-up study conducted by the former authors, galunisertib was also shown to reduce *COL1A1* mRNA expression in PCKS prepared from (fibrotic) human tissue, thereby confirming therapeutically relevant effects on fibrogenesis as well as established fibrosis (85). Additionally, in neither study were harmful effects observed for galunisertib, as the mitochondrial activity and morphological features of the tissue remained intact. Having said that, the authors only analyzed samples after an incubation of 48 h.

#### Imatinib

Imatinib is an inhibitor of the ABL1, KIT, and PDGFR tyrosine kinases (97). This small-molecule has been shown to target TGF-β and PDGF signaling in a SMAD-dependent and independent manner. It is frequently prescribed as an oral antineoplastic agent for treating chronic myeloid leukemia and gastrointestinal stromal tumors (97–99). Like LY2109761, imatinib has been shown to attenuate pulmonary and renal fibrosis in various models (85, 98, 100). Bigaeva et al. (85) showed that imatinib attenuated the early onset of fibrogenesis but not established fibrosis in PCKS prepared from human tissue (within 48 h). Imatinib also did not cause noticeable toxic effects, as assessed by histological evaluations and based on a lack of effects on mitochondrial activity. These findings are in line with Daniels et al. (98), who found out that imatinib reduced the number of resident interstitial fibroblasts and myofibroblasts, and reduced the expression and interstitial accumulation of collagen type III and IV as well as fibronectin deposition in rats. The exact mechanism-of-action, however, remains unclear, and it also remains unknown whether the effects are sufficient to attenuate fibrosis during NMP.

#### Nintedanib

Nintedanib is a small-molecule oral tyrosine kinase inhibitor that targets the VEGF, FGF, and PDGF receptors (101). It is used for the treatment of idiopathic pulmonary fibrosis as well as non-small-cell lung cancer (102, 103), and has been shown to inhibit EMT by suppressing TGF-β/SMAD signaling (104). Using PCKS prepared from non-fibrotic human tissue, Bigaeva et al. (81) showed that nintedanib significantly reduced mRNA expression of *COL1A1, SERPINH1, FN-1, IL-1B, IL-6*, and *IL-8* after 48 h of incubation. On top of that, COL1A1, HSP47, and α-SMA were significantly reduced on protein level, indicating strong anti-fibrotic effects. These results are in agreement with Liu et al. (101), who reported that nintedanib prevented phosphorylation of the PDGF, FGF, and VEGF receptors and reduced pro-inflammatory cytokine expression as well as macrophage infiltration. Although nintedanib seems promising due to its anti-inflammatory and anti-fibrotic effects in early fibrogenesis, it did not have therapeutic effects in PCKS prepared from fibrotic human tissue. However, this finding does not disqualify nintedanib as a promising candidate because it could still be useful during NMP.

#### Butaprost

Butaprost is a selective EP_2_ receptor agonist, which can be used to modulate the COX2/PGE2 system (86). This system has been implicated in numerous physiological processes that occur in the kidney, such as controlling hemodynamics and regulating salt levels. PGE2 produces these effects through activation of the prostanoid receptors EP_2_ and EP_4_, The expression of these receptors seems to be increased during renal injury and fibrogenesis, suggesting a protective role in fibrosis (86). To further explore the role of EP_2_ in renal fibrosis, Jensen et al. (86) tested butaprost in various models, including PCKS prepared from fibrogenic human tissue. The authors reported that butaprost mitigated EMT induced by TGF-β (i.e., by inhibiting SMAD2 phosphorylation, as demonstrated *in vitro*). They also showed that butaprost significantly reduced mRNA expression of *COL1A1, FN*, and *ACTA2*, as well as protein expression of α-SMA. These findings were confirmed *in vivo*, and it seems to be the case that butaprost has a direct effect on TGF-β/SMAD signaling, independent of the cAMP/PKA pathway. There was also no evident toxicity observed for butaprost.

### Biopharmaceuticals and Beyond

Apart from small-molecule drugs, no other candidates have been tested *ex vivo* with the purpose of attenuating fibrogenesis and/or fibrosis in kidney tissue. Therefore, it would be particularly useful to investigate other therapeutic modalities as well, including therapeutic proteins and monoclonal antibodies, or perhaps even more advanced approaches, such as cell-based therapies or (lipid-based) nanoparticles encapsulating nucleic acids for transiently silencing or enhancing the expression of specific genes. Combinatorial therapies, affecting various stages of renal injury and fibrogenesis, could also be developed. Some of the previously described modalities have already been tested in kidneys to attenuate IRI, but not with the focus on fibrosis (105, 106). Of course, there are some points to consider when developing such therapies. It is important to investigate the distribution kinetics of respective modalities in donor kidneys when administered during NMP. Cells, for instance, are unlikely to uniformly distribute through the tissue. The same applies to nanoparticles. Lastly, when supplementing perfusion solutions with therapeutic proteins and/or monoclonal antibodies, it is imperative to check whether shear stress affects their stability (e.g., denaturation, aggregation, etc.) (107).

## Concluding Remarks

IF/TA is one of the main causes of chronic graft failure after kidney transplantation, and without proper treatment, recipients are forced to resume dialysis, undergo re-transplantation, or suffer from premature death. One of the key processes in the onset of fibrosis is TGF-β1/SMAD signaling, providing an effective target for suppressing the onset of fibrosis. TGF-β receptor inhibitor galunisertib displayed clear therapeutic effects in early and late stage fibrosis (within 48 h). Galunisertib could therefore be a suitable candidate for suppressing the onset of fibrosis in a renal transplant setting. In the future, galunisertib should be tested in an *ex vivo* NMP setup using, for example, porcine kidneys. After that, the same approach can be applied to discarded human kidneys to establish whether this approach has therapeutic merit. Ultimately, the combination of oxygenated HMP and NMP supplemented with pharmacologically-active substances could lead to better long-term outcomes for patients, and therefore improve their quality of life.

## Author Contributions

LL and MR drafted the framework and reviewed the literature. LL prepared the manuscript and designed the figures. MR provided comments and revised the text. All authors critically reviewed the manuscript and approved this manuscript for publication.

## Conflict of Interest

The authors declare that the research was conducted in the absence of any commercial or financial relationships that could be construed as a potential conflict of interest.

## Publisher's Note

All claims expressed in this article are solely those of the authors and do not necessarily represent those of their affiliated organizations, or those of the publisher, the editors and the reviewers. Any product that may be evaluated in this article, or claim that may be made by its manufacturer, is not guaranteed or endorsed by the publisher.

## Abbreviations

ATP: Adenosine triphosphate
CIT: Cold ischemia time
DBD: Donation after brain death
DCD: Donation after circulatory death
DGF: Delayed graft function
ECM: Extracellular matrix
EMT: Epithelial-to-mesenchymal transition
EndMT: Endothelial-to-mesenchymal transition
HMP: Hypothermic machine perfusion
IF: Interstitial fibrosis
IL-1β: Interleukin 1 beta
IL-6: Interleukin 6
IRI: Ischemia and reperfusion injury
LD: Living donor
NMP: Normothermic machine perfusion
PCKS: Precision cut kidney slices
PDGF: Platelet-derived growth factor
PGE2: Prostaglandin E2
ROS: Reactive oxygen species
SCS: Static cold storage
TA: Tubular atrophy
TGF-β1: Transforming growth factor beta 1
TNF-α: Tumor necrosis factor alpha
UW-MP: University of Wisconsin machine perfusion
α-SMA: Alpha smooth muscle actin.
